# Gallium-modified gelatin nanoparticles loaded with quercetin promote skin wound healing *via* the regulation of bacterial proliferation and macrophage polarization

**DOI:** 10.3389/fbioe.2023.1124944

**Published:** 2023-01-26

**Authors:** Ning Yang, Nianyuan Shi, Zhou Yao, Hang Liu, Weinan Guo

**Affiliations:** ^1^ Xijing Hospital, Fourth Military Medical University, Xi’an, China; ^2^ The Key Laboratory of Biomedical Information Engineering of Ministry of Education, Xi’an Jiaotong University School of Life Science and Technology, Xi’an, China; ^3^ State Key Laboratory for Mechanical Behavior of Materials, Xi’an Jiaotong University, Xi’an, China; ^4^ Department of Dermatology, Xijing Hospital, Fourth Military Medical University, Xi’an, China

**Keywords:** Gallium, nanoparticles, Quercetin, wound healing, macrophage

## Abstract

**Background:** Wound healing is a complicated process involving multiple cell components and can help the re-establishment of the skin’s barrier function. Previous studies have pointed out that bacterial infection and sustained inflammatory reactions are the main causes of the delay of wound closure and scar formation during wound healing. The effect of current approaches for scar-free wound repair still faces many challenges, and alternative therapeutic methods are urgently needed to be established.

**Methods:** The basic characteristics of the new-designed nanoparticles were clarified through the characterization of the material. The biocompatibility of the nanoparticles, as well as its effect on fibroblast function, anti-bacterial capacity, inflammation suppressive role, and the underlying mechanism were further verified by a panel of biochemical assays *in vitro*. Ultimately, pre-clinical rat model was employed to testify its role in wound healing and scar formation *in vivo*.

**Results:** Firstly, gallium-modified gelatin nanoparticles loaded with quercetin was successfully established, displaying good biocompatibility and facilitative effect on fibroblast function. In addition, the nanoparticles showed prominent anti-bacterial and inflammation-suppressive effects. What’s more important, the nanoparticles could also induce the polarization of macrophages from M1 to M2 phenotype to exert its inflammatory inhibitory role through TGF-β/Smad signaling pathway. Ultimately, *in vivo* experiment showed that the nanoparticles could effectively promote wound repair and inhibit scar formation during the process of wound healing.

**Conclusion:** Taken together, the new nanoparticles have good anti-bacterial and anti-scar formation effects and great potential in the field of skin wound repair, which provides a promising therapeutic strategy for wound treatment.

## Introduction

Wound healing is a complicated process involving multiple cell components and can help the re-establishment of the skin’s barrier function (Gurtner et al., 2008). Impaired function of fibroblast, bacterial proliferation, and prolonged activation of inflammatory reactions at the wound site are the major reasons for delayed wound healing and excess scar formation (Morris et al., 2014; Nanditha and Kumar, 2022; Shams et al., 2022). Delayed wound healing and the resultant occurrence of hypertrophic scar (HS) not only leads to a reduction in cosmetic effects, but also might result in impaired function of adjacent joints, greatly affecting patients’ quality of life (QOL). Of note, HS has been identified as one of the major unaddressed functional and psychosocial challenges facing global health (Ogawa et al., 2021). Previously, several agents like corticosteroids, bleomycin and verapamil have been reported to ameliorate scarring during the process of wound healing (Giugliano et al., 2003; Yamamoto, 2006; Atiyeh, 2020). Nevertheless, since that skin wound healing is a complicated process with risks of infection, as well as the detailed mechanism underlying wound healing and scar formation remains far from understood, the effect of current approaches for scar-free wound repair still faces many challenges (Alster, 2003), and alternative therapeutic methods are needed to be established.

In recent years, accumulative evidence has demonstrated that some types of new biomaterials, such as hydrogel nanoparticles, are of great therapeutic potential in wound healing repair and anti-scar treatment. Hydrogel nanoparticles are an emerging biomaterial technology, displaying advantages including relatively larger coverage area, strong absorbability, strong slow-release capacity, and minor side effects, and have great application prospect in tissue repair, targeted drug delivery and regenerative medicine (Huang et al., 2022). It has also been reported that the injectable hydrogel nanoparticle preparation is suitable for closure of irregular wound and wound surface (Tariq et al., 2022), indicating the potential of hydrogel nanoparticle in repairing skin wound. Of note, impaired function of fibroblast, bacterial proliferation, and prolonged activation of inflammatory reactions are among the most important causes of the delay of wound healing and excessive scar formation (Morris et al., 2014; Nanditha and Kumar, 2022; Shams et al., 2022). Therefore, if any hydrogel nanoparticles preparation with the capacity to potentiate fibroblast function, suppress bacterial proliferation and inhibit excess inflammation can be developed, it will be an ideal method for wound treatment with simultaneous effects on promoting wound closure and inhibiting scar formation.

Gallium composite is an international emerging anti-bacterial material (Clarkin et al., 2019). The iron-eating property of bacteria is utilized to make the gallium ion that is highly similar to iron ion enter cells in place of iron, and then disrupt the iron metabolism of bacteria to reduce their survival rate to achieve the anti-bacterial effect (Wang et al., 2020). Of note, the anti-bacterial property of gallium ion has been verified in many studies (Qi et al., 2022; Zheng et al., 2022), and the Food and Drug Administration (FDA) also affirmed that gallium has good anti-bacterial activity and can be used in medicine (Rossato et al., 2022). While gallium ion-coated nanoparticles have been prepared in some studies and used to treat diseases like liver abscess and cancer (Xie et al., 2021; Yang et al., 2021). Their anti-bacterial effects on biological wound remain elusive. Quercetin is a flavonol compound among flavonoids, mainly exists in the form of glycosides, is widely distributed in multiple plants, fruits and traditional Chinese medicine and rick in red wine, and has anti-inflammatory, anti-oxidant, anti-bacterial, anti-cancer and anti-viral effects (Pinheiro et al., 2021). Some studies have suggested that the anti-inflammatory effect of quercetin is associated with its mechanisms of inhibiting the secretion of inflammatory factors and interfering with inflammatory signaling pathways (Chanjitwiriya et al., 2020; Dehghani et al., 2021). In previous studies, quercetin has been used to prevent peritoneal scar adhesion in abdominal surgery (Zeng et al., 2022). Nevertheless, its effect on suppressing sustained inflammation and preventing scar formation during skin wound healing warrants further investigation.

Inflammatory response is greatly implicated in the process of wound healing and scar formation, in which macrophage undergoing specific phenotypic and functional changes plays a cardinal role in all stages of the healing process. The dysregulation of the macrophages’ function is highly correlated with delayed wound healing and scarring (Sim et al., 2022). While the infiltration of the pro-inflammatory macrophage in the early phase of wound healing is necessary for the wound-healing progression, sustained existence of pro-inflammatory macrophages in remodeling phase could delay re-epithelialisation accompanied by an increased frequency of vascular leakage, immature granulation, and the persistence of neutrophils, which all contribute to delayed wound healing and increased scar formation (Ishida et al., 2008). Therefore, to suppress the infiltration of pro-inflammatory macrophage and induce macrophage polarization from M1 to M2 type in late phase is beneficial for wound healing.

In the present study, gallium-modified gelatin nanoparticles loaded with quercetin were firstly established, and its anti-bacterial and healing-promoting effects were testified *in vitro*. Then, its role in suppressing sustained inflammation and the underlying mechanism was investigated, focusing on the regulation of macrophage polarization. Furthermore, the therapeutic effect of this preparation on skin wound healing was examined in pre-clinical rats *in vivo*.

## Material and methods

### Agents, antibodies and animals

Gelatin (Gel), quercetin (QCT), gallium nitrate hydrate, and common chemical reagents were purchased from Sigma-Aldrich (United States). Dulbecco’s modified eagle medium (DMEM) and fetal bovine serum (FBS) were purchased from Gibco (United States). The following primary antibodies were purchased from Abcam (United States): Collagen 1 (COL1) (ab270993), Elastin (ab307151), Arginase 1 (Arg1) (ab203490), TGF beta Receptor II (TGFβR2) (ab259360), Smad2 (ab40855), p-Smad2 (ab280888). The following primary antibodies were purchased from Invitrogen (United States): CD206 (17-2061-82), CD86 (12-0862-82). TGFβR2 inhibitor LY2109761 were purchased from Selleck (United States). Cell viability/cytotoxicity detection kit were purchased from Solarbio (China). TNF-α, MCP-1, TGF-β3, IL-4 ELISA kit were purchased from eBioscience (United States).

Sprague-Dawley (SD) rats were purchased from Air Force Medical University. RAW264.7 cell line was obtained from National Collection of Authenticated Cell Cultures, The Academy of Sciences of China (SCSP-5036). *E. coli* and (ATCC 8739) and *S. aureus* (ATCC 6538) were obtained from the American Type Culture Collection (ATCC). All animal procedures were approved by the Experimental Animal Ethics Committee of Air Force Medical University.

### Synthesis of QCT@GNP-Ga

GNP was synthesized according to a previous protocol (Xu et al., 2013). Briefly, 0.1 g of gelatin (225 Bloom strength) was dissolved in 10 mL of deionized water at 50°C. The gelatin solution’s pH value was adjusted to 7.0 by 0.1 M NaOH. The nanoparticles forming procedure was conducted by adding acetone dropwise to the gelatin solution under continuous stirring. Then, 200 μL of 40% w/w glyoxal was added and mixed by stirring for 3 h at 100 g to crosslink the nanoparticles. 1 mM glycine was used to quench the surface-accessible aldehyde groups of glyoxal. The nanoparticles were obtained by centrifugation at 10,000 g for 20 min and re-suspended in PBS. QCT was added when the pH of the gelatin solution was adjusted to 7.0. The gallium ionic-GNPs were prepared by placing 3 wt% GNP solution in a grooved mold. When dialysis membrane (MWCO 3500 Da) was covered, 0.1 M Ga(NO_3_)_3_ aqueous solution was added. After soaking for 4 h, Ga3^+^-cross-linked GNPs were finally produced.

### Characterization of QCT@GNP-Ga

The chemical group alterations in the QCT@GNP and QCT@GNP-Ga were tested by a Fourier transform infrared (FTIR, Bruker, Germany) spectrometer with a wavenumber range of 4,000–500 cm^−1^. The morphologies of QCT@GNP and QCT@GNP-Ga samples were recorded by a transmission microscope (TEM, JEM-2100, JEOL, Japan).

### Drug release from QCT@GNP-Ga

The medium was prepared with 1 mg/mL lysozyme phosphate-buffered saline (PBS) solution (pH = 7.4, pH = 6.8). 0.5 g of QCT@GNP or QCT@GNP-Ga samples were soaked in 10 mL of the medium, agitating at 100 rpm and 37°C. At certain time intervals (12 h, 24 h, 36 h, 48 h, 60 h, 72 h, 84 h, and 96 h), a 3-mL volume was taken for measurement, and fresh medium was added to replenish. To remove the interference of the BSA, high-performance liquid chromatography was performed to test the concentration of QCT at 355 nm. The accumulated release was calculated to clarify the drug release profile and explore the drug release mechanism.

### Cell toxicity examination

The cells were seeded into 48-well plates at a density of 1.5 × 10^4^ cells/well and became adherent in 6 h. Discarded the medium and replaced with 10% of QCT@GNP or QCT@GNP-Ga extract. After 24, 48, and 72 h of co-culture, the cells were stained with calcein-AM/propidium iodide to observe the morphology and proliferation *via* confocal laser microscopy (Lecia, Germany).

### RNA extraction and qRT-PCR

Total RNA was extracted using EZNA Total RNA Kit II (OMEGA Bio-tek) and reverse transcription was performed using PrimeScript RTase (Takara Bio Inc.). The mRNA expression levels were measured with real-time quantitative reverse transcription PCR (qRT-PCR) using Premix Ex Taq (Takara) and standardized according to the endogenous control (β-actin) expression level. The cycle conditions were as follows: 95°C for 2 min, denaturation at 95°C for 5 s, annealing at 55°C for 10 s and finally extension at 72°C for 45 s. All the reactions were conducted in triplicate. The amplification and melt curves were used to identity the consistency of specific PCR product. The results were analyzed by using the 2^−ΔΔCT^ method.

### Protein extraction and immunoblotting analysis

After cells were lysed by RIPA buffer (Beyotime) added with PMSF (Beyotime), protein concentration was identified by the BCA method kit (Solarbio). Protein samples were collected using 10% SDS-PAGE (Beyotime) and transferred to the polyvinylidene fluoride membranes (PVDF, Millipore). After blocked with 5% skim milk for 1 h, the membrane was incubated with primary antibodies overnight at 4°C and then with the corresponding horse radish peroxidase (HRP)-conjugated secondary antibody (1:2000 dilution) for 1 h at RT. Finally, enhanced chemiluminescence substrate (ECL kit, Millipore) were used for visualization.

### Immunohistochemical staining

Scar tissues were obtained, soaked in 4% paraformaldehyde fixative, and then embedded by paraffin and finally cut into 4 μm slices. The following procedures were performed using biotin-streptavidin peroxidase method (SPlink Detection Kit, ZSGB-Bio). According to the manufacturer’s instruction, the paraffin-embedded slices were dewaxed, rehydrated using graded ethanol diluent, subjected to antigen restoration, incubated with 30% H_2_O_2_-CH_3_OH, and blocked with goat serum. Then the slices were incubated with the corresponding primary antibodies, followed by the biotinylated goat anti-rabbit IgG and HRP-conjugated streptomycin. Finally, Diaminobenzidine (ZSGB-Bio) was used for chromogenic reaction. The slices were observed using optical microscope (Olympus).

### Flow cytometry analysis

The cells treated with LPS and GNP-Ga or QCT@GNP-Ga were dissociated and resuspended. Fc block (Biosciences, United States) was used to prevent non-specific binding. All the detecting cells were stained with anti-CD86 and CD-206. Analysis was processed on a flow cytometer (BD Biosciences) with the FlowJo software (Tommy Digital Biology, Tokyo, Japan).

### ELISA

Cell supernatants were used to quantify the TNF-α, MCP-1, TGF-β3, and IL-4 with the detection limits of 7 pg/mL. Total protein was examined using a commercial kit (BCA protein assay kit, Pierce, Rockford, IL).

### Statistical analysis

Each experiment was performed at least three times, and statistical analyses of the data were performed using unpaired, two-tailed Student’s *t*-tests built into GraphPad Prism (GraphPad Software 8.0; San Diego, CA, United States). Data were presented as the mean ± S.D. *p* values of<0.05 were considered statistically significant.

## Results

### Characterization of QCT@GNP-Ga

**FIGURE 1:**
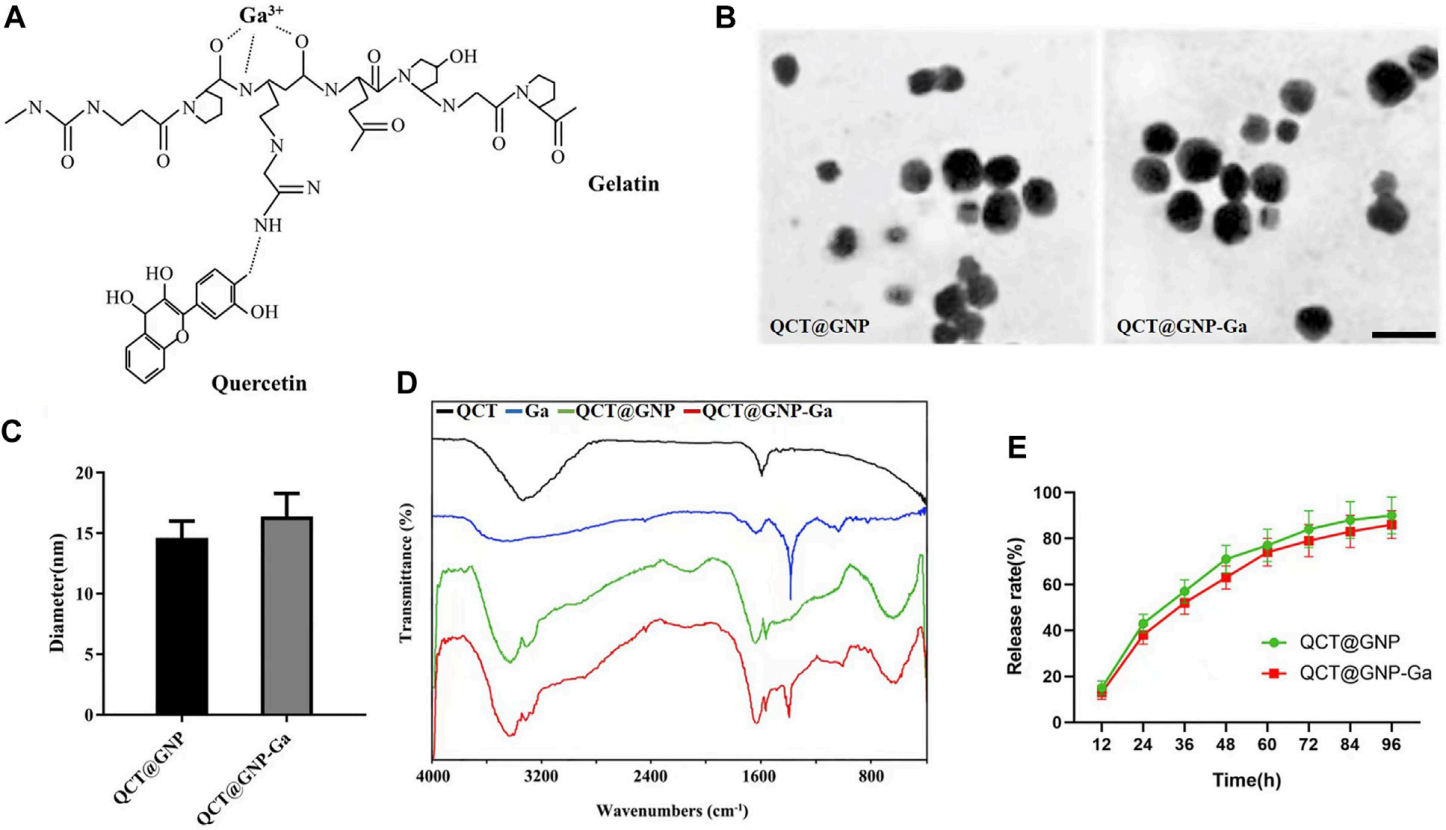
Characterization of QCT@GNP-Ga. **(A)** A schematic view of the design of gallium-modified gelatin nanoparticles loaded with quercetin. **(B)** Representative image TEM images of QCT@GNP and QCT@GNP-Ga, Scale bar = 20 nm. **(C)** Particle size of QCT@GNP and QCT@GNP-Ga. **(D)** FTIR spectra of QCT, Ga, QCT@GNP, and QCT@GNP-Ga. **(E)** QCT release from QCT@GNP and QCT@GNP-Ga.

The quercetin (QCT)-loaded gelatin nanoparticles (QCT@GNP) were prepared by dissolving, and then gallium ions were crosslinked onto the surface of nanoparticles by ion cross-linking to establish QCT@GNP-Ga (Figure 1A). Firstly, nanoparticles were observed under a transmission electron microscope (TEM), which showed that both QCT@GNP and QCT@GNP-Ga were of uniform and smooth spherical structures. The diameter of QCT@GNP and QCT@GNP-Ga was 14.6 ± 1.4 nm and 16.3 ± 1.9 nm, respectively (Figures 1B, C). Further, the molecular structure of the nanoparticle was detected by Fourier transform infrared spectrum. The results showed that the characteristic peak of gallium ion (Ga^3+^) was at 1,380 cm^−1^, while that of QCT was at 1,600 cm^−1^. The characteristic peaks of QCT@GNP were at 1,600 and 1,680 cm^−1^. Combined with the characteristics of the three above, the characteristic peaks of QCT@GNP-Ga were at 1,380, 1,600 and 1,680 cm^−1^. Of note, the characteristic peak of QCT@GNP at 1,600 cm^−1^ was shifted, indicating that the amido bonds in gelatin nanoparticles were strengthened under the action of gallium ion (Figure 1D). The controlled release of drugs by nanoparticles was further detected, which revealed that both QCT@GNP and QCT@GNP-Ga can be released stably in PBS solution for 96 h. The release curve of QCT@GNP-Ga is gentler (Figure 1E).

### Biocompatibility of QCT@GNP-Ga and its effect on function of fibroblasts

**FIGURE 2:**
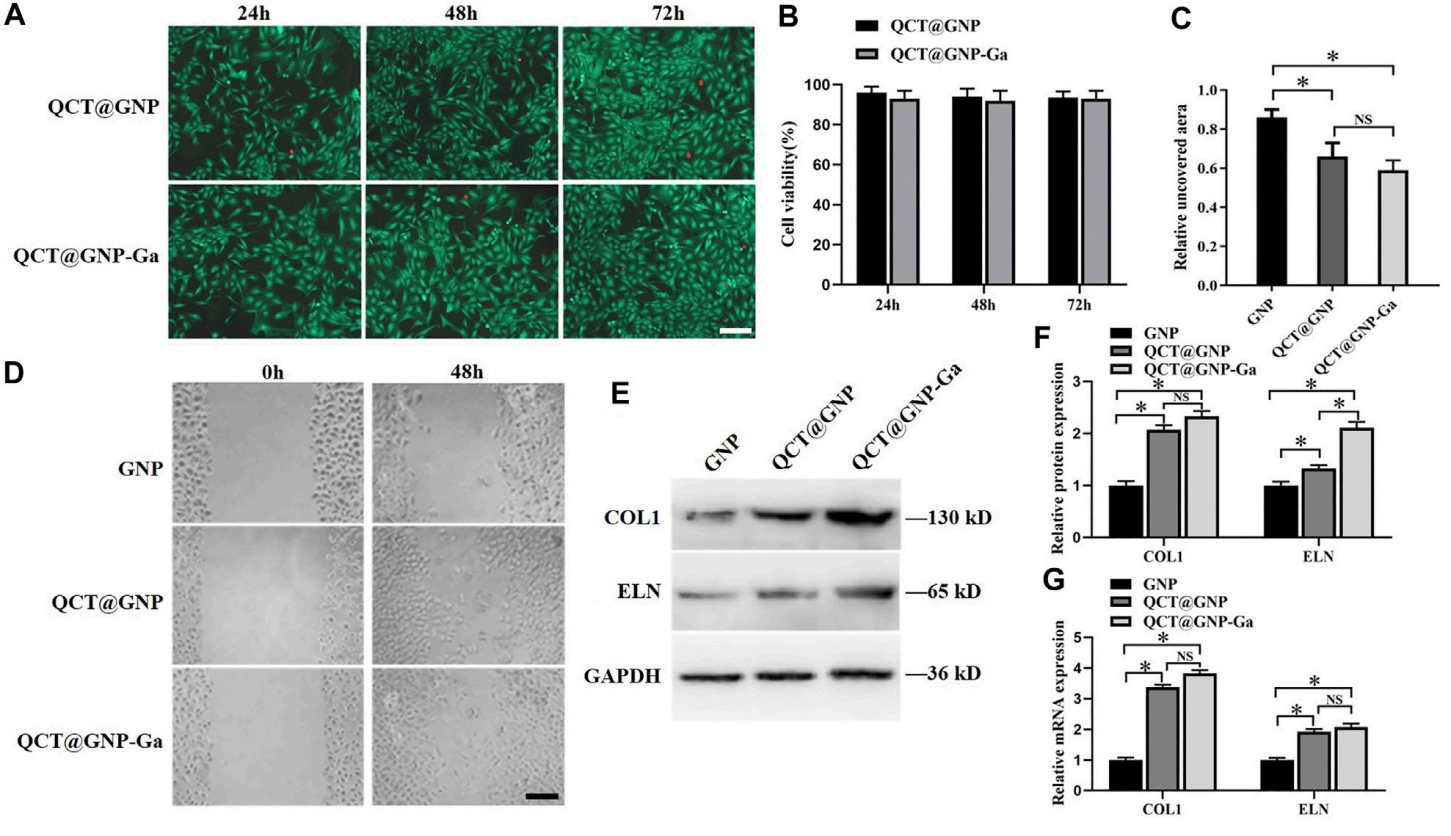
Effect of QCT@GNP-Ga on the cell function of primary fibroblasts. **(A)** Live/dead staining of fibroblasts treated with QCT@GNP or QCT@GNP-Ga. Scale bar = 100 μm. **(B)** Cell viability of fibroblasts treated with QCT@GNP or QCT@GNP-Ga for 24, 48, and 72 h. **(C)** The uncovered aera and **(D)** representative images of wound-healing assay of fibroblasts treated with GNP, QCT@GNP or QCT@GNP-Ga. **(E)** Western blot analysis and **(E)** statistical results of COL1, ELN protein expressions in fibroblasts treated with GNP, QCT@GNP or QCT@GNP-Ga. **(G)** qRT-PCR analysis of COL1, ELN mRNA expressions in fibroblasts treated with GNP, QCT@GNP or QCT@GNP-Ga for 48 h. Data are shown as mean ± SD, ^*^ *p* < 0.05 compared with GNP group.

QCT@GNP-Ga was added to the medium of the mouse primary skin fibroblasts cultured *in vitro* to observe its effect on the function of fibroblasts. Firstly, the results of cell survival/death staining showed that there was no significant difference in the cell survival between the QCT@GNP group and the QCT@GNP-Ga group, indicating that both QCT@GNP and QCT@GNP-Ga have good biocompatibility with mouse fibroblasts and are not cytotoxic (Figures 2A, B). Besides, cell wound-healing assay showed that the cell migration in the QCT@GNP group and the QCT@GNP-Ga group at 48 h was significantly greater than that in the GNP group. However, the difference between the QCT@GNP group and the QCT@GNP-Ga group was insignificant (Figures 2C, D). Human skin is constructed with many proteins, among which Type I collagen (COL1) and elastin (ELN) that are derived from fibroblasts and constitute extracellular matrix (ECM) play a key role in providing strength and elasticity to the human skin and body (Lee et al., 2022). The expressions of COL1 and ELN can well reflect the ability of fibroblast to produce ECM that is related to process of wound healing and scar formation (Bui et al., 2022). qPCR and WB analysis showed that 48 h after the treatment with QCT@GNP or QCT@GNP-Ga, both the mRNA and protein levels of COL1 and ELN were significantly higher than those in the GNP group (Figures 2E–G). The above results showed that QCT@GNP-Ga has no cytotoxicity to mouse fibroblasts and can promote fibroblast migration and matrix secretion.

### Anti-bacterial activity of QCT@GNP-Ga

**FIGURE 3:**
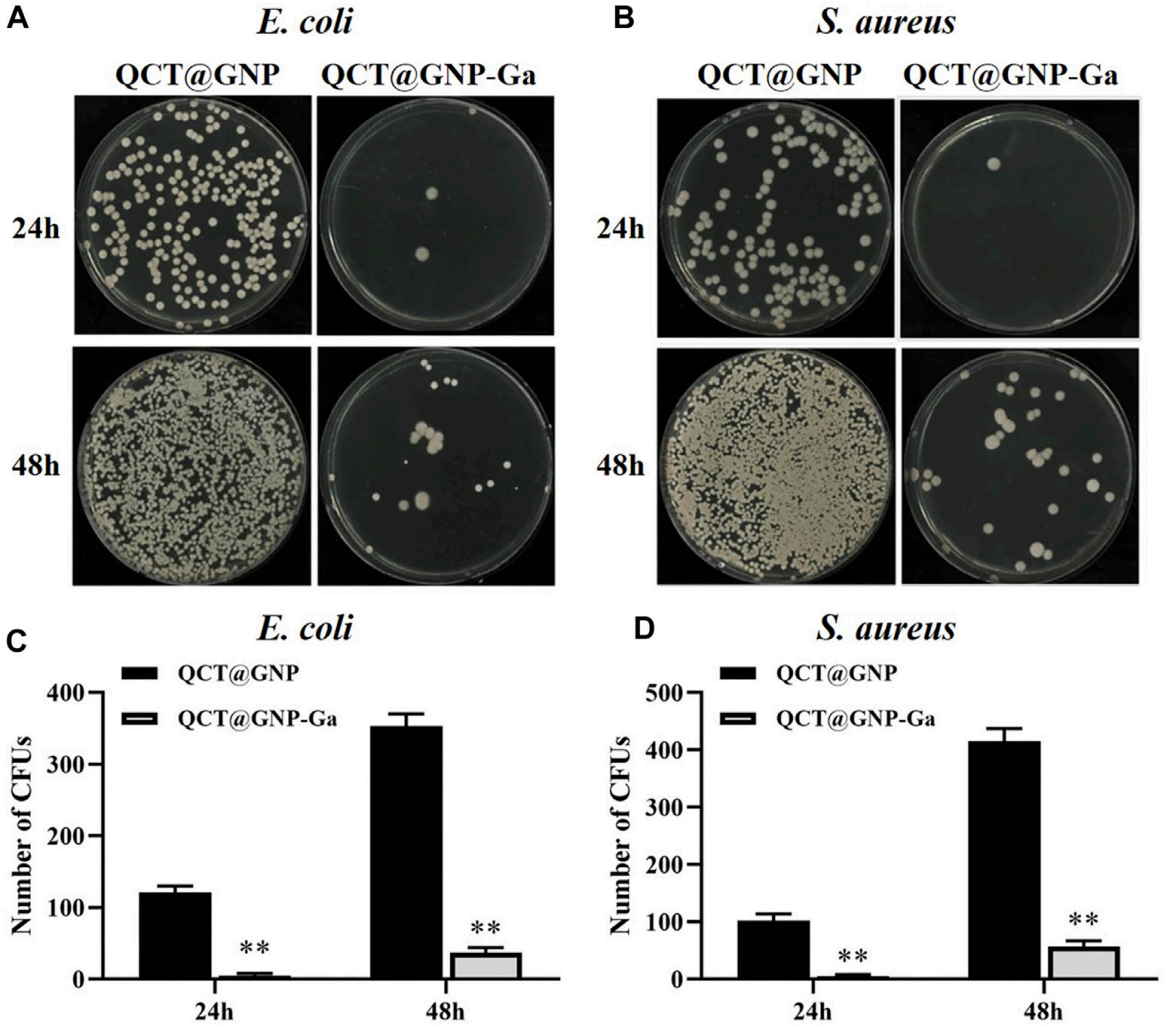
Anti-bacterial activity of QCT@GNP-Ga. **(A, B)** Images and **(C, D)** statistical results of colony forming unit (CFU) of *E. coli* and *S. aureus* treated with QCT@GNP or QCT@GNP-Ga. Data are shown as mean ± SD, ^*^ *p* < 0.05 compared with QCT@GNP group.

Since that bacterial proliferation at the wound site is one of the major reasons for delayed healing and abnormal scarring (Frykberg and Banks, 2015; Zhang et al., 2022), and Gallium and Quercetin are documented of anti-bacterial property (Hu et al., 2022; Qin J. et al., 2022), we went on to investigate whether QCT@GNP-Ga exerted great anti-bacterial effect. The surface of the agarose medium was coated with QCT@GNP-Ga and then inoculated with *S. aureus* and *E. coli* to observe its anti-bacterial activity. After 24 h of constant temperature culture at 37°C, many bacterial colonies were observed on the culture dish in the QCT@GNP group, while only a few bacterial colonies were observed in the QCT@GNP-Ga group. At 48 h, the culture dish in the QCT@GNP was covered with bacterial colonies, while only a few bacterial colonies were formed in the QCT@GNP-Ga group (Figures 3A, B). Further statistics analysis showed that the number of bacterial colonies formed in the QCT@GNP-Ga group at either 24 or 48 h was significantly smaller than that in the QCT@GNP group. The same results were obtained for *S. aureus* and *E. coli* (Figures 3C, D).

### Induction of macrophages from M1 to M2 phenotype by QCT@GNP-Ga

**FIGURE 4:**
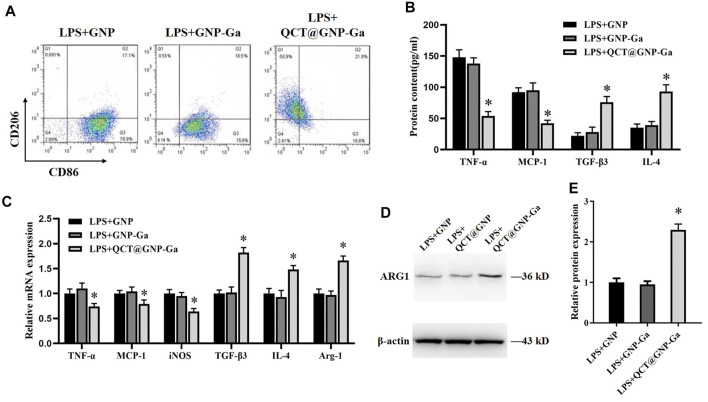
Induction of macrophages from M1 to M2 phenotype by QCT@GNP-Ga. **(A)** Representative images of FCM analysis of LPS induced RAW264.7 cells treated with GNP, GNP-Ga, or QCT@GNP-Ga. **(B)** EILSA analysis of TNF-α, MCP-1, TGF-β3, and IL-4 protein expressions in LPS induced RAW264.7 cells treated with GNP, GNP-Ga, or QCT@GNP-Ga. **(C)** qPCR analysis of TNF-α, MCP-1, iNOS, TGF-β3, IL-4, Arg-1 mRNA expressions in LPS induced RAW264.7 cells treated with GNP, GNP-Ga, or QCT@GNP-Ga. **(D)** Western blot analysis and **(E)** statistical results of ARG1 protein expressions in LPS induced RAW264.7 cells treated with GNP, GNP-Ga, or QCT@GNP-Ga. Data are shown as mean ± SD, ^*^ *p* < 0.05 compared with LPS + GNP group.

It has been demonstrated that a proper inflammatory response is required for skin wound repair (Xu et al., 2010), while prolonged inflammation leads to pathological healing especially scarring (Morris et al., 2014). Macrophages are the most functionally diverse cells for generation and resolution of inflammation by phenotype polarization. Classically-activated macrophages (M1) secrete high levels of pro-inflammatory cytokines like IL-1β, IL-6, and TNF-α to exhibit pro-inflammatory properties (Koh and DiPietro, 2011), whereas alternatively-activated macrophages (M2) that secret high level IL-10 and TGF-β and low-level IL-12 play a role in resolution of inflammation (Gensel and Zhang, 2015). High population of macrophages could lead to sustained inflammation that might delay the process of wound healing (Guo et al., 2016). Therefore, we wondered whether QCT@GNP-Ga played a role in the regulation of macrophage polarization. To this end, mouse RAW264.7 macrophages were cultured *in vitro* and induced to polarize to M1 by LPS. Afterwards, these macrophages were treated with QCT@GNP-Ga for 5 days to observe its effect on the polarization of mouse macrophages. Flow cytometry analysis revealed that, after the induction by LPS, CD86 was highly-expressed in cells, while CD206 was merely expressed. After the treatment with QCT@GNP-Ga, the expression of CD86 in RAW264.7 cells decreased, while the expression of CD206 increased, indicating the polarization of macrophages from M1 to M2 phenotype (Figure 4A). Further ELISA assay showed that, in response to LPS stimulation, the expression of TNF-α and MCP-1 was significantly lower in the QCT@GNP-Ga group than that in the GNP group and GNP-Ga group, while the expression of TGF-β3 and IL-4 was prominently increased in QCT@GNP-Ga group (Figure 4B). In addition, qRT-PCR analysis showed that in response to LPS stimulation, the mRNA levels of TNF-α, MCP-1 and iNOS were significantly lower in the QCT@GNP-Ga group than those in the GNP group and GNP-Ga group, while the mRNA levels of TGF-β3, IL-4 and Arg-1 were significantly higher than those in the GNP group and GNP-Ga group (Figure 4C). Similarly, immunoblotting analysis proved that the expression of Arg-1 was prominently increased after the treatment with QCT@GNP-Ga (Figures 4D, E). Taken together, these results demonstrated that QCT@GNP-Ga could induce the phenotype switch of macrophages from M1 to M2, so that sustained inflammation could be mitigated to facilitate the process of wound healing.

### Regulation of polarization of macrophages by QCT@GNP-Ga through TGF-β/Smad pathway

**FIGURE 5:**
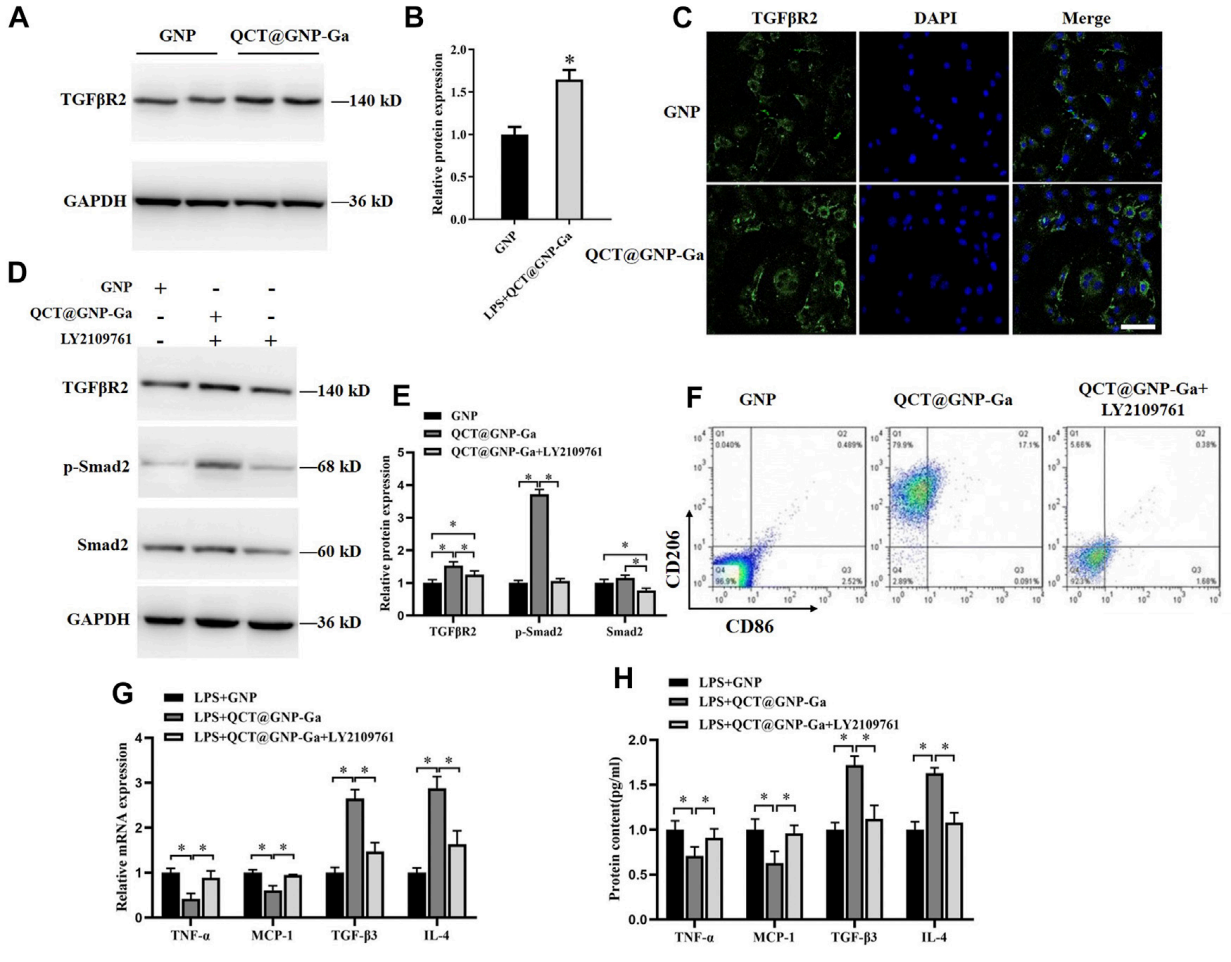
Regulation of polarization of macrophages by QCT@GNP-Ga through TGFβ/Smad pathway. **(A)** Western blot analysis, **(B)** statistical results, and **(C)** immunofluorescent staining analysis of TGFβR2 protein expressions in RAW264.7 cells treated with GNP or QCT@GNP-Ga. **(D)** Western blot analysis and **(E)** statistical results of TGFβR2, p-Smad2, and Smad2 protein expressions in RAW264.7 cells treated with GNP or QCT@GNP-Ga. **(F)** Representative images of FCM analysis of RAW264.7 cells treated with GNP or QCT@GNP-Ga or LY2109761. **(G, H)** Relative expression and secretion of TNF-α, MCP-1, TGF-β3 and IL-4 after the treatment with GNP or QCT@GNP-Ga or with LY2109761 in LPS-stimulated macrophage. Data are shown as mean ± SD, ^*^ *p* < 0.05, Scale bar = 20 μm.

Thereafter, we went on to elucidate the mechanism underlying the role of QCT@GNP-Ga in the regulation of macrophages polarization. To this end, QCT@GNP-Ga was added to the medium of the RAW264.7 cells cultured *in vitro* for 48 h, after which the expressions of related signaling pathways were detected. It was found that the protein expression of TGFβR2 was increased after the treatment with QCT@GNP-Ga compared with control GNP group (Figures 5A, B). In consistent, immunofluorescence staining analysis also showed that the expression of TGFβR2 was enhanced after the treatment with QCT@GNP-Ga (Figure 5C). In line with the upregulation of TGFβR2 after QCT@GNP-Ga treatment, the phosphorylation of Smad2 was also increased, indicating the activation of TGFβ-Smad signaling by QCT@GNP-Ga, and this effect could be effectively blocked by TGFβR2 inhibitor LY2109761 (Figures 5D, E). Flow cytometry analysis revealed that the expression of CD206 in cells increased after the treatment with QCT@GNP-Ga, while its expression was significantly reduced by the inhibitor LY2109761 (Figure 5F). In addition, while QCT@GNP-Ga treatment induced prominent downregulation of the expression and secretion of TNF-α and MCP-1, as well as the upregulation of the expression and secretion of TGF-β3 and IL-4, co-treatment with TGFβR2 inhibitor LY2109761 was capable of reversing this alteration trend (Figures 5G, H). Therefore, these results suggested that the activation of TGF-β/Smad pathway mediated the role of QCT@GNP-Ga in macrophages polarization from M1 to M2.

### *In vivo* repair effect of QCT@GNP-Ga on rat skin wound

**FIGURE 6:**
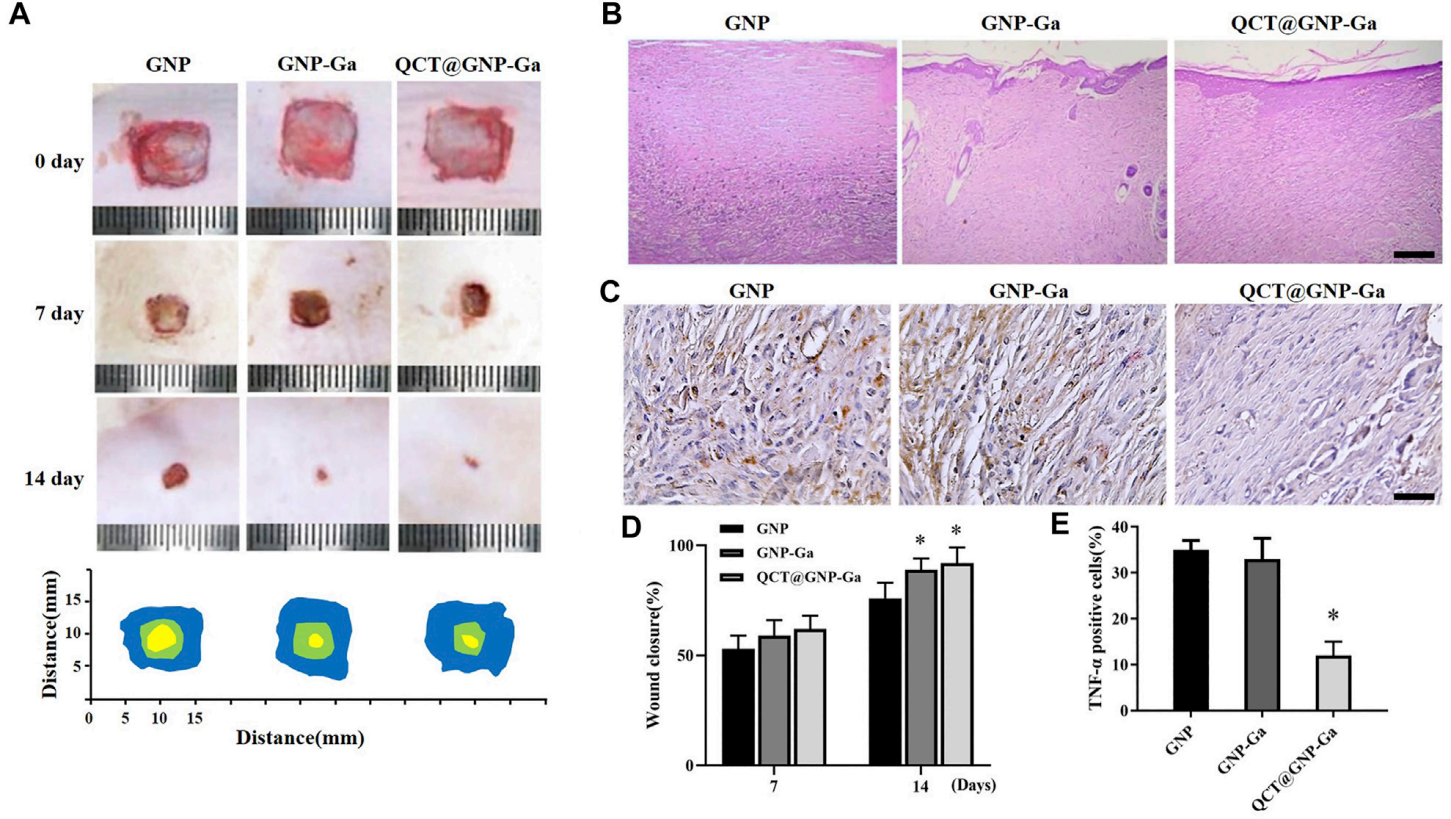
*In vivo* repair effect of QCT@GNP-Ga on rat skin wounds. **(A)** Representative images of wound healing covered by GNP, GNP-Ga or QCT@GNP-Ga from 0 to 14 days and **(D)** quantitative results. **(B)** Representative HE staining images of tissue from healing wounds of GNP, GNP-Ga, or QCT@GNP-Ga group, Scale bar = 100 μm. **(C)** Representative TNF-α immunohistochemical staining images and **(E)** quantitative results of tissue from healing wounds of GNP, GNP-Ga or QCT@GNP-Ga group, Scale bar = 20 μm. Data are shown as mean ± SD, ^*^ *p* < 0.05 compared with GNP group.

Given that QCT@GNP-Ga could simultaneously potentiate fibroblast function, exert anti-bacterial property, and induce macrophages polarization from M1 to M2 to suppress sustained inflammation, we proposed that QCT@GNP-Ga might play a cardinal role in facilitating wound healing and restraining scar formation *in vivo*. A skin wound model was established on the back skin of SD rats, and then the injectable material for repairing skin wound prepared with the gelatin hydrogel loaded with QCT@GNP-Ga. After 14 days of injection, the average rates of wound healing in both the GNP-Ga group and the QCT@GNP-Ga groups were higher than that in the GNP group (Figures 6A, D). The results of HE staining showed that many fibrous scars were formed at wound locations in the GNP group, and local fibrocytes proliferated and were arranged in a compact and disordered manner. In contrary, the scar tissues in the GNP-Ga group were partially reduced, and were further reduced in the QCT@GNP-Ga group (Figure 6B). Concurrent immunohistochemical staining analysis showed that the TNFα-positive cells in the control group were distributed in scar tissues, while the TNFα-positive cells in the QCT@GNP-Ga group were significantly reduced (Figures 6C, E), indicating that the reduction of scar tissues was associated with the inhibition of inflammatory reactions. Taken together, QCT@GNP-Ga exerts great potential in facilitating wound healing and restraining scar formation in pre-clinical rat model.

## Discussion

With the improvement of people’s living standard, the attention to efficient wound healing paralleled with reduced scar formation has been gradually increased. In case of an open skin wound, it is often accompanied with bacterial attachment, causing wound infection and resultant severe scars (Leng et al., 2022). In addition, the impaired wound-healing function of fibroblast and sustained inflammatory reactions also restrain the process of wound closure and increase the incidence or severity of scar (Morris et al., 2014; Shams et al., 2022). In the present study, a novel anti-bacterial drug-loaded hydrogel nanoparticle was developed, which could effectively facilitate wound closure, inhibit bacterial proliferation, and ameliorate scar formation, which was related to its role in regulating macrophage polarization, providing a promising choice for the treatment of wound.

Since that nanoparticles have multiple advantages including high drug loading capacity, good biocompatibility, and the ability of the controlling drug release, they have been widely employed in investigations on tissue repair. As a drug carrier or antibacterial active substance, nanoparticles are applied to skin damage repair by mixing with hydrogel to form composite hydrogel (Sun et al., 2021; Burdusel et al., 2022; Qin P. et al., 2022). Silver particles, titanium oxide, gentamicin-containing nanoparticles, curcumin-containing nanoparticles, and other composite hydrogels have been confirmed to have anti-bacterial and wound healing-promoting effects (Javanmardi et al., 2018; Zahiri et al., 2020; Xiang et al., 2022). In recent years, gallium ion has been found to be advantageous in the field of antibacterial materials (Vinuesa and McConnell, 2021). Due to the unique anti-microbial mechanism of gallium, it can overcome the problem of resistance to traditional antibiotics, such as the prevention of drug absorption due to the permeability of bacterial cell wall. Gallium ions can imitate the metabolic pathway of iron ions to promote cell absorption, whereas cannot be reduced like iron ions (Kurtuldu et al., 2022). Therefore, the proliferation of bacteria is inhibited when the oxidation-reduction process necessary for the synthesis of the DNA and protein of bacteria is interrupted. In addition to the recognized broad-spectrum bactericidal activity of gallium ions, small doses of gallium ions can also suppress inflammatory reactions (Ma and Fu, 2010; Goss et al., 2018). Besides, some studies have demonstrated that gallium ions can also promote collagen synthesis and cell migration, thus facilitating the process of wound healing (Kircheva and Dudev, 2019; Castilla-Amoros et al., 2020). In this study, our data proved that the gallium ion-modified gelatin nanoparticles were efficient in promoting the wound-healing function of fibroblast, suppressing bacterial proliferation and inhibiting inflammatory reactions, providing an experimental foundation for wound treatment.

Quercetin is flavonoid rich in a variety of foods and has multiple biological characteristics, including anti-inflammatory reaction, anti-oxidative and some other properties (Liu et al., 2019; Mi et al., 2022). Some studies have pointed out that quercetin can reduce the generation of TNF-α, IL-6 and IL-1 in mononuclear U937 cells induced by LPS (Okoko and Oruambo, 2009). In addition, quercetin can inhibit the expressions of inflammation-related genes in RAW264.7 macrophages (Cui et al., 2019). The study by Gupta et al. (2016) proved that quercetin can effectively ameliorate the eosinophilic airway inflammation in allergic asthma. Moreover, Guan et al. (2021) demonstrated that quercetin can relieve rheumatoid arthritis (RA) by promoting the apoptosis of neutrophils. Supplementary to these previous reports, our data for the first time proved that quercetin was also effective in inhibiting the inflammatory reactions during skin scar formation. Inflammatory response participates in wound healing and scar formation, and macrophages that undergo phenotypic and functional changes play a critical role. The persistent existence of pro-inflammatory M1 macrophage in regional skin could prominently delay would healing (Ishida et al., 2008; Sim et al., 2022). To suppress the infiltration of pro-inflammatory macrophage and induce macrophage polarization from M1 to M2 type in late phase is beneficial for wound healing and reduced scar formation. To be specific, quercetin regulated the polarization of macrophages towards M2 through TGF-β/Smad signaling pathway to inhibit inflammation and thereby reduce scar formation, further supporting that the intervention of macrophages polarization might be a useful strategy to reduce scar formation risk during the process of wound healing.

## Conclusion

In the present study, a new gallium ion-modified drug-loaded hydrogel nanoparticle was developed aiming to ameliorate infection and scar formation during the process of wound healing. The anti-bacterial, healing-promoting and scar formation-inhibitory effects of the nanoparticles were verified both *in vitro* and *in vivo*, with particular emphasis on the regulation of macrophages polarization *via* TGF-β/Smad pathway to suppress sustained inflammatory reactions at wounds. Our data demonstrated that gallium-modified gelatin nanoparticles loaded with quercetin could promote skin wound healing *via* the regulation of bacterial proliferation and macrophage polarization, and this preparation might be a promising choice to treat wound and suppress scar formation simultaneously, which needs further confirmation by clinical trial in the future.

## Data Availability

The raw data supporting the conclusion of this article will be made available by the authors, without undue reservation.
